# A multidimensional structure of digital anxiety in older people with scale development and validation

**DOI:** 10.3389/fpsyg.2026.1942275

**Published:** 2026-10-29

**Authors:** Zhemin Zhu, Fangfei Qiao

**Affiliations:** 1 School of Educational Science, Beihua University, Jilin, China; 2 Jilin Open University, Changchun, China

**Keywords:** digital anxiety, digital inclusion, older people, scale development, technostress

## Abstract

**Inroduction:**

As digital technologies become increasingly embedded in everyday life, older people may experience persistent psychological challenges when engaging with complex and evolving digital systems. This study examines digital anxiety as a multidimensional construct in later life and develops a corresponding measurement scale. Drawing on technostress theory and related gerontological perspectives, digital anxiety is conceptualized as comprising four dimensions: operational learning anxiety, technological risk anxiety, ability-based exclusion anxiety, and privacy and institutional anxiety.

**Methods:**

Data were collected from three independent samples. The main sample (*N* = 691) was split for exploratory and confirmatory factor analyses. Additional model comparisons were conducted to examine alternative factor structures. Reliability and validity were evaluated through internal consistency, test–retest reliability, convergent and discriminant validity, and associations with external constructs.

**Results:**

Exploratory and confirmatory factor analyses supported a correlated four-factor structure with acceptable model fit. Additional model comparisons indicated that the substantial covariance among ability-based exclusion anxiety, operational learning anxiety, and privacy and institutional anxiety could also be represented by a partial second-order factor, whereas technological risk anxiety remained a separate first-order dimension. The scale demonstrated high internal consistency, satisfactory test–retest reliability, acceptable convergent validity, and generally supportive but not unequivocal evidence of discriminant validity. Criterion-related validity was supported through associations with technostress-related constructs and weak correlations with life satisfaction.

**Discussion:**

The findings provide a psychometrically promising instrument for examining digital anxiety among older people. The results support the multidimensional conceptualization of digital anxiety while highlighting the need for further examination of the relationships among its constituent dimensions.

## Introduction

The World Health Organization (WHO) estimates that the global population aged 60 and above will reach 1.4 billion by 2030 and 2.1 billion by 2050 (World Health Organization, 2025). Population ageing is occurring alongside another structural transformation: the rapid digitization of everyday life. Across healthcare, transportation, finance, and public services, activities once accessible through face-to-face interaction are increasingly mediated by smartphones, mobile applications, QR codes, biometric authentication, and algorithm-driven systems. As essential services become digitally mediated, older people’s ability and willingness to navigate digital systems may shape their access to healthcare, transportation, financial resources, and opportunities for social participation.

Frailty is increasingly understood in relation to the interaction between individuals’ capacities and the demands of their social and physical environments. From this perspective, barriers to accessing essential services and maintaining social participation may be relevant to efforts to prevent or delay frailty. Yet the traditional “digital divide” framework, which primarily emphasizes inequalities in access and skills (van Dijk, 2006), does not fully capture the psychological experiences that may shape older people’s engagement with digital systems. Older people may experience persistent tension, uncertainty, and loss of control when using digital technologies, particularly when interfaces are complex, rules change rapidly, or potential risks are unclear. These experiences may accumulate into digital anxiety, which is associated with avoidance of online services and disengagement from digitally mediated activities (Nimrod, 2018). Such avoidance may restrict access to essential services and reduce opportunities for social interaction, civic participation, and social connection (Seifert, 2020). In this way, digital anxiety may represent a psychological barrier to the service access and social participation that support older people’s resilience and are relevant to frailty prevention.

Despite increasing recognition of digital anxiety as a critical factor shaping older people’ adaptation to digital environments, existing research remains limited in two important respects. First, most studies conceptualize digital anxiety as a unidimensional construct or examine it through related concepts such as computer anxiety or technostress (Heinssen et al., 1987; Tarafdar et al., 2007). Such approaches often fail to capture the layered and evolving nature of anxiety experiences in contemporary digital contexts, where concerns may arise not only from operational difficulties but also from perceived risks, self-efficacy deficits, and institutional distrust. Second, there is a lack of measurement instruments specifically designed for older populations. Many existing scales are developed for students or working adults and may not adequately reflect the lived experiences, cognitive characteristics, and usage contexts of older users.

To address these gaps, the present study aims to develop and validate a multidimensional scale of digital anxiety tailored to older people. Drawing on prior research on computer anxiety, technostress, digital anxiety, and technophobia concerns (Bozionelos, 2001; Ragu-Nathan et al., 2008; Kim et al., 2021; Nimrod, 2018), this study conceptualizes digital anxiety as a structured construct encompassing four interrelated dimensions: operational learning anxiety (OL), technological risk anxiety (TR), ability-based exclusion anxiety (AE), and privacy and institutional anxiety (PI). This framework represents four conceptually related domains concerning operational difficulty, perceived risk, competence-related self-evaluation, and trust in digital systems. It thus provides a measurement foundation for research on how digital inclusion can be incorporated into frailty-prevention strategies, including efforts to make essential services more accessible and to sustain older people’s participation in community life.

## Conceptualization and scale development

### Theoretical background

Early work on computer anxiety conceptualized anxiety as fear, tension, and avoidance arising in actual or anticipated interaction with computers, with particular emphasis on operational difficulty, task failure, and perceived incompetence (Simonson et al., 1987). Research on technophobia reached similar conclusions, showing that discomfort toward technology among older people is associated not only with fear of devices themselves but also with broader avoidance tendencies in digital life (Nimrod, 2018). However, this line of research is still largely centered on concrete use situations and does not fully capture the broader social and institutional pressures of digitalized living.

A second strand of scholarship has moved from device-specific anxiety toward broader concerns about digital transformation. Digitalisation anxiety has been defined as a generalized tension and discomfort regarding the emergence and societal integration of new technologies, thus extending anxiety beyond discrete episodes of use to anticipatory concerns about ongoing technological change (Pfaffinger et al., 2021). Similarly, digital phobia in older people is framed as pervasive anxiety toward broad digital environments, not just individual interfaces, reflecting age-related factors and global digitalization effects. These perspectives are valuable because they foreground uncertainty, loss of control, and future-oriented apprehension. Yet they remain either too broad or too population-unspecific to explain how anxiety is concretely experienced in later life, where declining learning confidence, accumulated risk perceptions, and dependence on digitally mediated services interact in distinctive ways.

A third line of research, more closely tied to ageing, suggests that older people’ technology-related anxiety is inseparable from questions of competence, participation, and exclusion. Studies have shown that older people’ use of technology depends not only on access but also on confidence, perceived ability, and emotional responses to digital interaction (Czaja et al., 2006; Kim et al., 2023). At the same time, research on digital exclusion indicates that older people’ digital difficulties are embedded in wider structural inequalities, including reduced access to services, weakened participation, and feelings of being left behind in a digital society (Wang and Chen, 2015). This means that older people’ digital anxiety should not be treated as a unidimensional emotional disposition. Rather, it appears to involve at least operational strain, perceived risk, competence-related self-doubt, and concerns about exclusion or loss of control.

The existing studies establish that older people are particularly vulnerable to negative emotional responses in digital environments, but the available concepts remain fragmented. Some focus too narrowly on operational interaction, some are too broad and anticipatory, and others identify important correlates such as self-efficacy or exclusion without integrating them into a coherent anxiety framework. What remains insufficiently specified is a theory-driven account that links technology-related strain to the age-specific conditions of later life and that can support the development of distinct measurement domains.

Gerontological theories provide an important developmental context for understanding these age-specific conditions. Lawton and Nahemow’s (1973) competence–environmental press model proposes that adaptation depends on the fit between individuals’ competencies and the demands imposed by their environments. Applied to digital contexts, this perspective helps explain why complex or rapidly changing technologies may be experienced differently depending on older people’ perceived capabilities and available resources. Similarly, the model of selective optimization with compensation (Baltes and Baltes, 1990) emphasizes that adaptation in later life involves adjusting goals, optimizing available resources, and compensating for constraints. These perspectives are useful for understanding heterogeneity in older people’ responses to digital environments, but they do not specify the technology-specific demands that constitute the content of digital anxiety. For this purpose, technostress theory provides a complementary and more proximal framework for identifying specific digital stressors and organizing the domains of the present scale.

### Conceptual development

For older people, digital anxiety should therefore be conceptualized as a distinct and multidimensional phenomenon rooted in the conditions of later-life digital participation. In contemporary digital environments, older people are not only asked to learn new interfaces, but also to navigate unstable procedures, assess potential risks, protect personal data, and maintain social participation under conditions of unequal competence. Prior research suggests that older people’ digital participation is shaped not only by structural barriers, such as limited access or poorly designed digital environments, but also by person-level psychological constraints, including low confidence in using digital technologies, anxiety about making mistakes, perceived technological risk, and a diminished sense of control during digital interaction (Kim et al., 2023). In the present study, these psychological constraints are distinguished from environmental or structural barriers because they refer to individuals’ subjective appraisals and emotional responses to digital demands rather than to the external conditions themselves.

Against this gerontological background, technostress theory was used as the proximal organizing framework for specifying the content domains of digital anxiety. This choice does not imply that technostress theory substitutes for theories of aging. Rather, the two perspectives serve complementary functions: aging theories help explain how older people adapt to changing personal resources and environmental demands, whereas technostress theory specifies technology-related stressors such as complexity, insecurity, and uncertainty (Tarafdar et al., 2007; Ragu-Nathan et al., 2008; Ayyagari et al., 2011). Computer anxiety, self-efficacy, and digital exclusion perspectives further inform particular aspects of the construct, but none alone provides the same differentiated account of technology-specific demands required for the present multidomain scale development.

These stressors are not themselves psychological constructs; rather, they describe structural properties of digital environments. In the context of older people, these structural demands are experienced and internalized as qualitatively different forms of anxiety, shaped by age-related cognitive, social, and experiential conditions. It therefore offers a more suitable organizing framework for conceptualizing digital anxiety as a multidomain psychological response rather than a single affective reaction.

Specifically, techno-complexity and aspects of techno-overload are primarily translated into operational learning anxiety, as older people encounter difficulties in understanding interfaces, remembering procedures, and adapting to frequent updates. Techno-uncertainty and techno-insecurity, which reflect unpredictability and perceived vulnerability in digital environments, are more directly associated with technological risk anxiety, characterized by concerns about potential negative consequences such as fraud or misuse, particularly when these outcomes are perceived as difficult to control or reverse. Ability-based exclusion anxiety represents a related but conceptually distinct domain concerning self-evaluative doubts about competence and perceived fit within digital environments (Compeau et al., 1999; Kim et al., 2023). Finally, techno-insecurity and aspects of techno-invasion, particularly those related to loss of control over personal information and opaque system governance, are reflected in privacy and institutional anxiety, which concerns distrust toward the rules, data practices, and decision-making mechanisms embedded in digital systems, as well as the perceived lack of control over their outcomes (Smith et al., 1996; Ayyagari et al., 2011).

### Conceptual derivation of four dimensions

Guided by this framework, the present study conceptualizes digital anxiety among older people as comprising four related but distinct dimensions: operational learning anxiety, technological risk anxiety, ability-based exclusion anxiety, and privacy and institutional anxiety. These dimensions are not proposed as interchangeable expressions of a single diffuse worry. Rather, each represents a specific domain in which anxiety may arise during later-life digital participation.

Operational learning anxiety refers to the tension, confusion, and frustration older people experience when learning to use or actually using digital devices, platforms, or applications. This dimension originates from research literature on computer anxiety. Many studies have indicated a strong correlation between anxiety and anticipated errors, task failure, and difficulty performing technology-related operations (Heinssen et al., 1987; Knowles and Hanson, 2018). Repeated operational difficulties may therefore be associated with heightened anxiety during digital interaction. Furthermore, this dimension is also closely related to the technological complexity component of technological stress—that is, the technology is perceived as difficult to understand and requires skills that the user believes they do not possess (Tarafdar et al., 2007). In later life, such anxiety may be amplified by frequent interface changes, multi-step procedures, and the cumulative strain of having to repeatedly relearn digital operations. Operational learning anxiety is distinguished by its focus on direct interaction with technology itself rather than on the anticipated consequences of that interaction.

Risk represents another important source of anxiety for older people, especially given the prevalence of electronic fraud today. Technological risk dimension is derived primarily from the insecurity and uncertainty components of technostress and includes fears related to fraud, misinformation, privacy leakage, data misuse, financial loss, and irreversible operational errors (Tarafdar et al., 2007; Ayyagari et al., 2011; Pfaffinger et al., 2021). For older people, such concerns may be especially salient because digital actions are often experienced as high-stakes and difficult to reverse. Technological risk anxiety and operational learning anxiety are related but distinct. Conceptually, technological risk anxiety differs from operational learning anxiety in that its object is not the difficulty of performing digital tasks, but the anticipated negative outcomes that may follow from using technology incorrectly or under uncertain conditions. In other words, operational learning anxiety concerns whether one can complete the task, whereas technological risk anxiety concerns what may happen if something goes wrong.

Ability-based exclusion anxiety is another psychological state. The ability-based exclusion anxiety refers to anxiety arising when individuals interpret their perceived digital incompetence as a sign of personal inadequacy and reduced fit within a digitalized environment. This dimension builds on research showing that perceived technological competence is closely tied to self-efficacy and willingness to engage with digital tools (Compeau and Higgins, 1995), but it extends beyond competence judgment alone. In a highly digitalized society, digital ability increasingly functions as a marker of autonomy, normal participation, and social legitimacy. In the present framework, this dimension captures situations in which perceived difficulty with digital technologies is accompanied by self-doubt about one’s capability to participate effectively in digital environments. This dimension therefore connects individual competence concerns with perceived limitations in engaging effectively in digital environments (Li and Kostka, 2024; Han et al., 2025). The ability-based exclusion anxiety is not identical to low self-efficacy. Self-efficacy concerns one’s capability judgment, whereas ability-based exclusion anxiety concerns the broader implications for perceived capability and participation.

Privacy and institutional anxiety refers to anxiety arising from uncertainty about the transparency, trustworthiness, and controllability of system-governed digital processes. It is informed by research on information privacy concerns, particularly apprehension about how organizations collect, store, use, and manage personal information, but extends beyond privacy in a narrow technical sense. In the present framework, this dimension also includes concerns about identity verification, transaction procedures, opaque platform rules, data management, and digital processes whose operation or consequences may be difficult for users to understand, challenge, or reverse. Privacy and institutional anxiety is conceptually adjacent to, but distinct from, technological risk anxiety. Technological risk anxiety is defined primarily by the anticipated harmful consequences of digital technology use, such as fraud, misuse, financial loss, or other adverse outcomes. By contrast, privacy and institutional anxiety is defined primarily by the perceived characteristics of the systems and organizations governing digital interactions, including whether their procedures are transparent, trustworthy, and controllable. Thus, similar digital situations may evoke both forms of anxiety, but the two dimensions differ in their primary object of concern: anticipated harmful outcomes in technological risk anxiety versus the governance and controllability of system-level processes in privacy and institutional anxiety.

The four dimensions are conceptualized as related domains rather than as sequential stages of a developmental process. Although operational difficulty and competence-related self-doubt may be theoretically associated, the present cross-sectional design does not establish temporal ordering or causal progression among these forms of digital anxiety. Their developmental relationships should therefore be examined in longitudinal research.

## Materials and methods

### Item generation

Item development was guided by the multidimensional framework proposed in the preceding section and by the actual content domains reflected in older people’ digital experiences. Rather than directly adopting any single existing instrument, the present study used an integrative item-generation strategy in which technostress served as the overarching organizing framework, while specific item content was informed by adjacent strands of measurement research on computer anxiety, computer self-efficacy, information privacy concerns, and digital exclusion. Accordingly, the final scale was designed to capture four related but distinguishable domains of digital anxiety in later life: operational learning anxiety, technological risk anxiety, ability-based exclusion anxiety, and privacy and institutional anxiety.

Item content was developed by translating the four conceptual domains into situations that older people commonly encounter in everyday digital use. At the most immediate level, items for operational learning anxiety were informed by research on computer anxiety and techno-complexity, which emphasizes tension and confusion arising from operational difficulty, task failure, and insufficient mastery (Heinssen et al., 1987; Simonson et al., 1987; Li and Kostka, 2024). Accordingly, these items focused on experiences such as adapting to software updates, remembering procedural steps, and navigating complex application functions. Beyond difficulties in task execution, digital participation may also evoke anxiety about what could happen when technology is used incorrectly or under uncertain conditions. Items for technological risk anxiety therefore drew on the insecurity and uncertainty components of technostress and on research concerning digitalisation anxiety (Tarafdar et al., 2007; Ayyagari et al., 2011; Pfaffinger et al., 2021). The resulting items addressed concerns about fraud, information misuse, rapid technological change, and potentially irreversible mistakes. A related domain concerns how older people evaluate their own capability in digital contexts. Building on research on computer self-efficacy, which links perceived technological competence with confidence, persistence, and emotional responses to technology use (Bandura, 1997; Han et al., 2025), items for ability-based exclusion anxiety captured self-doubt and perceived inadequacy associated with difficulties in completing common digital tasks, such as video calling, understanding application functions, and participating in online activities. Finally, some sources of anxiety extend beyond individual task performance or perceived competence to the broader systems within which digital activities occur. Drawing on the literature on information privacy concerns (Smith et al., 1996), items for privacy and institutional anxiety addressed concerns about the trustworthiness and controllability of digital systems, including mobile payment and online banking, management of personal information, opaque system procedures, and uncertainty surrounding digital records and data. Accordingly, items in this dimension focused on anxiety concerning how digital systems and organizations manage personal information, transactions, verification procedures, and other consequential processes. Although some of these situations may also involve perceived technological risk, items were assigned to PI when their primary focus was the transparency, trustworthiness, or controllability of the system or institutional process rather than the anticipated harmful outcome itself. This distinction was used to differentiate PI from technological risk anxiety during the conceptual review of the retained items.

Following the initial item-generation process, an initial pool consisted of 30 candidate items designed to provide broad coverage of the four proposed content domains (8 OL items, 7 TR items, 9 AE items, and 6 PI items). Each item was initially mapped to the domain it was primarily intended to represent, although some items were deliberately retained at this stage despite potential conceptual overlap with adjacent domains. The items were independently reviewed by five experts with relevant disciplinary backgrounds, who evaluated their relevance, clarity, and appropriateness for older people and provided suggestions for improving item wording and meaning. Based on this feedback, ambiguous, overly complex, or potentially misleading expressions were revised to improve conceptual clarity and accessibility. The revised 30-item pool was then subjected to exploratory factor analysis. Item reduction was conducted iteratively: items with primary factor loadings below 0.40 were removed, and the EFA was rerun after item removal until a stable and interpretable factor solution was obtained. This process resulted in a final set of 21 items representing the four dimensions of digital anxiety. All items were rated on a five-point Likert scale ranging from 1 (“strongly disagree”) to 5 (“strongly agree”) (Figure 1).

**Figure 1:**
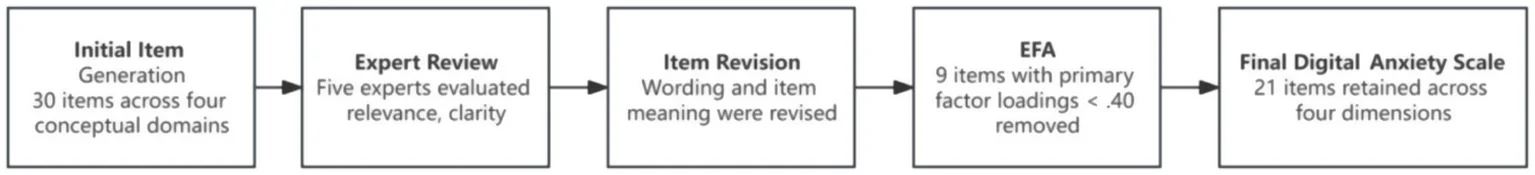
Item generation and reduction process for the Digital Anxiety Scale.

### Participants

Data were collected across three rounds of questionnaire administration for different stages of scale development and validation. All participants were older people aged 60 years or above. The first round was used for the main psychometric analyses, including exploratory factor analysis and confirmatory factor analysis. A total of 819 questionnaires were distributed, and 691 valid responses were retained, yielding a valid response rate of 84.3%. In this sample, 215 participants were male and 476 were female; 326 were aged 60–64 years and 365 were aged 65 years or above.

The second round was conducted for the test–retest assessment. In total, 326 questionnaires were distributed, of which 296 were valid, corresponding to a valid response rate of 90.80%. Among these participants, 85 were male and 211 were female; 138 were aged 60–64 years and 158 were aged 65 years or above.

The third round was used to examine criterion-related validity. A total of 317 questionnaires were distributed, and 290 valid responses were obtained, resulting in a valid response rate of 91.48%. In this subsample, 68 participants were male and 222 were female; 119 were aged 60–64 years and 171 were aged 65 years or above.

All participants were informed about the purpose of the study, and informed consent was obtained prior to their participation. Participation was voluntary and all responses were collected anonymously.

### Methods

Data collection was conducted in three stages corresponding to scale development, test–retest reliability, and criterion validity assessment. Participants completed the questionnaires anonymously, and all responses were screened for completeness before analysis.

Data analysis was carried out in several steps. Exploratory factor analysis (EFA) was conducted using the first subsample (*n* = 413) with principal axis factoring and Promax oblique rotation because the proposed dimensions were theoretically expected to be correlated. Sampling adequacy was assessed using the Kaiser–Meyer–Olkin (KMO) measure and Bartlett’s test of sphericity. Factor retention was determined by considering eigenvalues greater than 1, inspection of the scree plot, and the conceptual interpretability of the resulting solution. Items with primary factor loadings below 0.40 were removed during the item-reduction process, while cross-loadings and conceptual relevance were also examined when evaluating the final solution.

Second, confirmatory factor analysis (CFA) was performed using the independent validation subsample (n = 278) to test the four-factor structure identified in the EFA. Model fit was evaluated using multiple indices, including the chi-square statistic (χ^2^), the normed chi-square (χ^2^/df), the comparative fit index (CFI), the root mean square error of approximation (RMSEA), and the standardized root mean square residual (SRMR).

Finally, the reliability was assessed using Cronbach’s alpha coefficients for internal consistency and test–retest correlations for temporal stability based on the second sample. The convergent and discriminant validity were examined. Convergent validity was assessed using composite reliability (CR) and average variance extracted (AVE). Discriminant validity was first evaluated using the Fornell–Larcker criterion by comparing the square root of AVE for each construct with the corresponding inter-construct correlations. Given the relatively high correlations among several dimensions, discriminant validity was further examined using the heterotrait–monotrait ratio of correlations (HTMT). HTMT values below 0.90 were considered indicative of adequate discriminant validity. In addition, 95% bootstrap confidence intervals for the HTMT estimates were obtained using 5,000 bootstrap resamples; discriminant validity was considered further supported when the confidence interval did not include 1.00. Criterion-related validity was tested by examining the associations between digital anxiety and techno-complexity, techno-uncertainty, life satisfaction in the third sample.

The derived data supporting the findings of this study are available in the DCD repository (DOI: 10.17632/pcf4rgmdk2.1), and competing-model analyses were conducted in R version 4.5.2.

## Results

### Reliability

The overall Cronbach’s alpha coefficient for the 21-item scale was 0.907, indicating excellent internal consistency. At the dimensional level, all four subscales exceeded the recommended threshold of 0.70, demonstrating satisfactory reliability. In addition, test–retest reliability was assessed using an independent sample (*n* = 296) over a two-week interval. The coefficients ranged from 0.768 to 0.885, suggesting acceptable temporal stability of the scale. These results indicate that the scale demonstrates both strong internal consistency and satisfactory temporal stability.

### Construct validity

To evaluate the psychometric properties of the scale, the valid sample from the first wave (*N* = 691) was randomly split into two subsamples. Specifically, about 60% of the sample (*n* = 413) was used for exploratory factor analysis (EFA), while about the remaining 40% (*n* = 278) was used for confirmatory factor analysis (CFA). This procedure follows common practice in scale development to ensure that the factor structure identified in the exploratory stage can be independently validated.

For the final 21-item solution, the KMO measure was 0.943 and Bartlett’s test of sphericity was significant, χ^2^(210) = 4984.784, *p* < 0.001, confirming the suitability of the data for factor analysis. Four factors had initial eigenvalues greater than 1, whereas the fifth eigenvalue decreased to 0.738. Inspection of the scree plot also indicated a substantially flatter pattern after the fourth factor. Together with the conceptual interpretability of the solution, these results supported retention of four factors. The four-factor solution accounted for 56.779% of the variance after extraction. The complete Pattern Matrix is presented in Table 1.

**Table 1:** Rotated factor loadings (EFA).

Item	AE	OL	PI	TR
I worry about making mistakes when trying new functions.	**0.700**	−0.026	0.229	−0.128
I feel that digital technologies are not suitable for me.	**0.695**	−0.012	0.076	0.012
I feel nervous about using AI technologies.	**0.671**	0.104	0.155	−0.105
I feel uneasy about not knowing how to use video calls.	**0.665**	−0.048	0.184	−0.161
I lack confidence in my ability to use digital technologies effectively.	**0.654**	0.125	−0.212	0.241
I often question whether learning these technologies is worth the effort.	**0.612**	−0.097	−0.048	0.277
I find the steps required to use apps too complex.	**0.577**	0.090	−0.044	0.176
Learning new digital skills requires a lot of effort.	**0.559**	0.205	−0.009	0.034
I worry that I cannot keep up with new technologies as AI develops.	−0.035	**0.862**	−0.047	0.014
I feel stressed when I think about learning how to download and install new software.	−0.017	**0.843**	0.031	−0.035
I feel anxious when I need to find a specific function in a new app.	0.137	**0.669**	0.069	−0.130
I worry about the safety of links or QR codes.	−0.099	**0.575**	0.178	0.085
I feel that technological advancements are too fast for me to learn.	0.300	**0.524**	−0.031	0.045
I quickly forget how to use certain digital functions, even after being taught.	0.399	**0.455**	−0.003	0.000
I worry about being scammed when using mobile payments or online banking.	0.115	−0.055	**0.842**	−0.050
I worry that my personal data may be leaked by apps.	0.029	0.014	**0.593**	0.252
I worry about making mistakes when using smartphones or computers.	0.037	0.191	**0.545**	0.154
I feel uneasy about using biometric technologies such as facial recognition or fingerprints.	0.073	0.187	**0.439**	−0.027
I feel unsafe when entering personal information online.	−0.208	0.067	0.170	**0.779**
I worry that AI may misuse my personal data.	0.138	−0.216	0.156	**0.633**
I find it hard to keep up because digital technologies change so quickly.	0.100	0.090	−0.163	**0.593**

AE, ability-based exclusion anxiety; OL, operational learning anxiety; PI, privacy and institutional anxiety; TR, technological risk anxiety. The bold values represent steps.

Particular attention was given to the distinction between PI and TR because these domains contain conceptually adjacent content. The four PI items showed primary loadings of 0.439–0.842 on the PI factor, whereas their loadings on TR ranged from −0.050 to 0.252. Conversely, the three TR items loaded 0.593–0.779 on the TR factor, with loadings on PI ranging from −0.163 to 0.170. Thus, although the two dimensions share some thematic content, the observed loading pattern supports retaining their current empirical assignments. Overall, the EFA results provide initial support for the four-factor structure and indicate that the items generally showed stronger empirical loadings on their designated factors, although some conceptual overlap remained between adjacent domains. One item showed a relatively small separation between its two highest pattern coefficients (Item 14, 0.455 on Factor 2 and 0.399 on Factor 1). This item was retained because its primary loading exceeded the prespecified 0.40 criterion and because it contributed theoretically relevant content to the corresponding domain. The remaining items showed clearer loading patterns.

The four-factor structure was further examined using confirmatory factor analysis (CFA) with the independent validation subsample. The model showed an acceptable, although not uniformly strong, fit to the data, χ^2^(183) = 536.27, *p* < 0.001, χ^2^/df = 2.93, CFI = 0.903, TLI = 0.889, RMSEA = 0.083, 90% CI [0.075, 0.092], and SRMR = 0.051. The CFI and SRMR were within commonly used ranges for acceptable fit, whereas the TLI fell slightly below the conventional 0.90 benchmark and the RMSEA indicated a moderate level of approximation error. Thus, the CFA results provide qualified rather than unequivocal support for the proposed four-factor structure. All four dimensions demonstrated acceptable internal consistency and convergent validity. Composite reliability values ranged from 0.746 to 0.901, and average variance extracted (AVE) values ranged from 0.502 to 0.593. To examine the relationships among the four first-order dimensions, standardized latent correlations were obtained from the CFA model and are presented in Table 2.

**Table 2:** Latent correlations among the four dimensions of digital anxiety.

Construct	AE	OL	PI	TR
AE	1.00	0.85	0.89	0.68
OL	0.85	1.00	0.82	0.56
PI	0.89	0.82	1.00	0.73
TR	0.68	0.56	0.73	1.00

AE, ability-based exclusion anxiety; OL, operational learning anxiety; PI, privacy and institutional anxiety; TR, technological risk anxiety.

As shown in Table 2, the four dimensions were moderately to strongly correlated, with latent correlations ranging from 0.56 to 0.89. The strongest association was observed between AE and PI (r = 0.89), followed by AE and OL (r = 0.85) and OL and PI (r = 0.82). By comparison, TR showed relatively weaker associations with the other dimensions, ranging from 0.56 to 0.73. This pattern indicates substantial shared variance among AE, OL, and PI, while TR appears comparatively more distinct. Given the magnitude of several inter-factor correlations, discriminant validity was examined further using the Fornell–Larcker criterion and the heterotrait–monotrait ratio of correlations (HTMT).

Discriminant validity was first examined using the Fornell–Larcker criterion. The square roots of AVE were 0.730 for ability-based exclusion anxiety (AE), 0.767 for operational learning anxiety (OL), 0.770 for privacy and institutional anxiety (PI), and 0.708 for technological risk anxiety (TR). The Fornell–Larcker criterion was not satisfied for several construct pairs. Specifically, the latent correlations between AE and OL (r = 0.847), AE and PI (r = 0.887), and OL and PI (r = 0.818) exceeded the corresponding square roots of AVE. The correlation between PI and TR (r = 0.725) also slightly exceeded the square root of AVE for TR. These results indicate substantial shared variance among several dimensions and provide only limited support for discriminant validity based on the Fornell–Larcker criterion alone.

Given these findings, discriminant validity was further evaluated using the heterotrait–monotrait ratio of correlations (HTMT) with bootstrap confidence intervals. HTMT values ranged from 0.579 to 0.856, and all six construct-pair estimates were below the 0.90 criterion. Specifically, the HTMT values and their 95% bootstrap confidence intervals were 0.826 [0.764, 0.882] for AE–OL, 0.856 [0.796, 0.909] for AE–PI, 0.673 [0.561, 0.773] for AE–TR, 0.829 [0.757, 0.893] for OL–PI, 0.579 [0.452, 0.699] for OL–TR, and 0.704 [0.573, 0.829] for PI–TR. None of the confidence intervals included 1.00. Taken together, these results provide additional support for the empirical distinctiveness of the four dimensions, although the relatively high HTMT values for AE–PI, OL–PI, and AE–OL indicate substantial overlap among these constructs. The substantial correlations among several first-order factors also motivated the subsequent examination of a possible higher-order factor structure.

### Criterion-related validity

To examine criterion-related validity, external criteria were selected to represent both theoretically related and theoretically distinct constructs. Specifically, two dimensions derived from the technostress creators framework—techno-complexity (TC) and techno-uncertainty (TU)—were used as related external criteria because they capture perceived difficulty in understanding and adapting to digital technologies, which is conceptually proximal to digital anxiety (Tarafdar et al., 2007). To ensure contextual relevance, the original items were carefully adapted to reflect everyday digital scenarios encountered by older people while preserving their original conceptual meaning. In contrast, the Satisfaction With Life Scale (SWLS) was employed as a theoretically distal criterion to test whether the newly developed scale could be distinguished from a broad evaluative indicator of global well-being (Pavot and Diener, 1993). Pearson correlations were then calculated between the four dimensions of the newly developed Digital Anxiety Scale and the three external criteria. The result is shown in Table 3.

**Table 3:** Pearson correlations among observed subscale scores and external criteria.

Variable	AE	OL	PI	TR	TC	TU	LS
AE	1						
OL	0.700**	1					
PI	0.751**	0.719**	1				
TR	0.666**	0.575**	0.718**	1			
TC	0.754**	0.675**	0.665**	0.743**	1		
TU	0.548**	0.498**	0.512**	0.592**	0.628**	1	
LS	0.152**	0.042	0.080	0.088	0.125*	0.185**	1

AE, ability-based exclusion anxiety; OL, operational learning anxiety; PI, privacy and institutional anxiety; TR, technological risk anxiety; TC, techno-complexity; TU, techno-uncertainty; LS, life satisfaction. *p < 0.05. **p < 0.01.

As shown in Table 3, all four dimensions were positively and significantly correlated with techno-complexity (rs = 0.665–0.754, ps < 0.001) and techno-uncertainty (rs = 0.498–0.592, ps < 0.001). These coefficients were moderate to high in magnitude and in the expected direction, indicating that higher digital anxiety was associated with stronger perceptions of technological complexity and uncertainty. The correlations with life satisfaction were uniformly weak (rs = 0.042–0.152). The generally low magnitude of these correlations indicates that the present scale is distinguishable from a broad global evaluation of life satisfaction, thereby offering additional evidence of discriminant criterion validity.

### Additional model comparisons

The relatively high latent correlations among AE, OL, and PI suggested that these three dimensions might share a broader common component, whereas TR showed comparatively weaker associations with the other dimensions. To examine this possibility, additional model comparisons were conducted. In addition to the correlated four-factor model, we tested a partial second-order model in which AE, OL, and PI loaded onto a common second-order factor, while TR was retained as a separate first-order factor. We also tested a more parsimonious two-factor model in which all items from AE, OL, and PI were specified to load directly onto a single first-order factor, with TR retained as the second factor (Figure 2).

**Figure 2:**
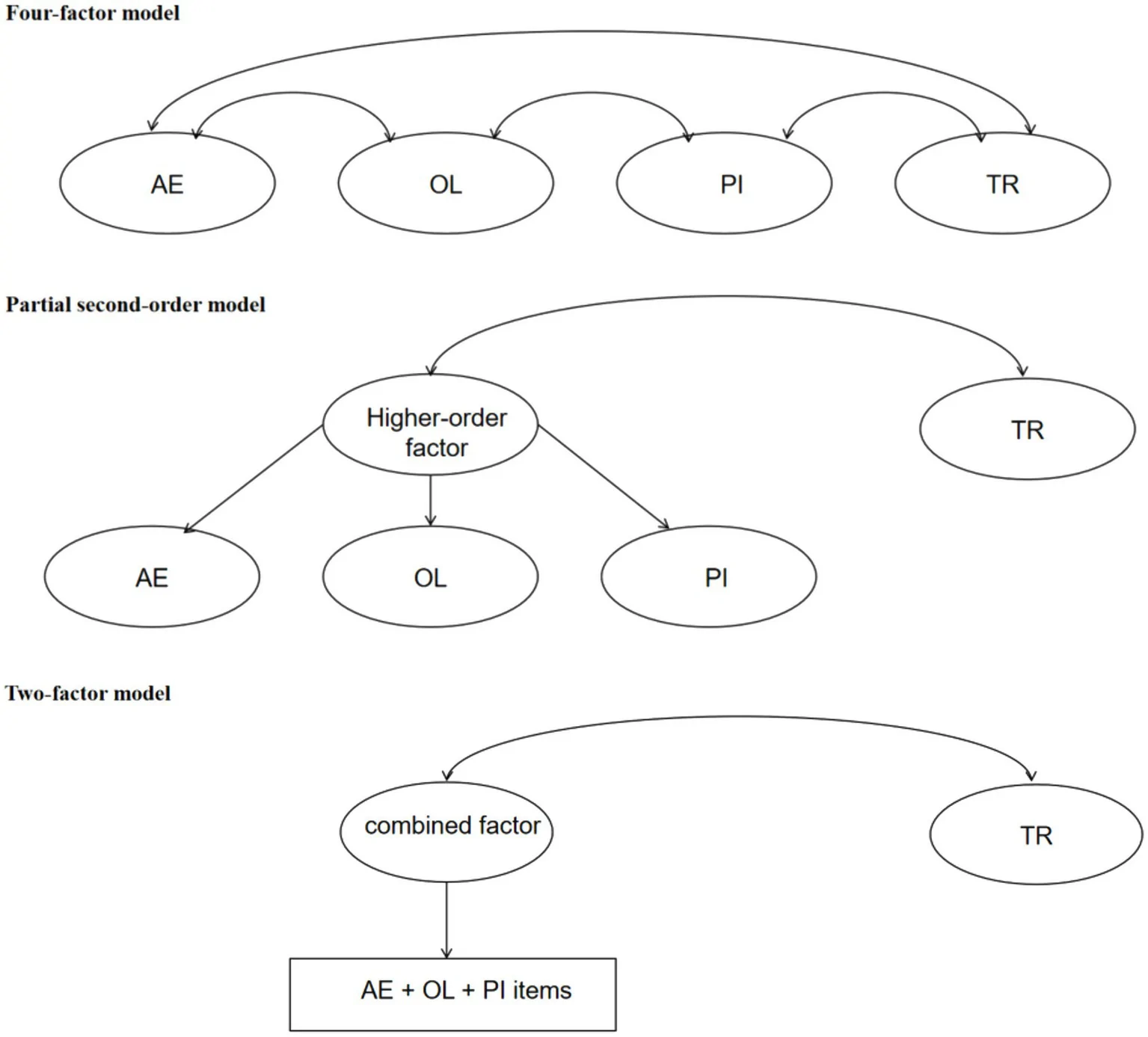
Competing measurement models.

The competing models were estimated in R using the lavaan package with maximum-likelihood estimation. Latent variables were scaled using the fixed-factor identification method (std.lv = TRUE), such that latent (residual) variances were fixed to 1.0 and factor loadings were freely estimated rather than identifying the factors by fixing a marker loading to 1. In the partial second-order model, AE, OL, and PI were specified as first-order factors loading on a common higher-order factor, whereas TR remained a separate first-order factor. The higher-order factor was allowed to correlate with TR. Latent covariances were explicitly specified rather than added automatically.

The fit statistics for these alternative models are presented in Table 4.

**Table 4:** The results of model comparisons.

Model	χ^2^	df	χ^2^/df	CFI	TLI	RMSEA	90% CI	SRMR	AIC	BIC
Four-factor	536.27	183	2.93	0.903	0.889	0.083	[0.075, 0.092]	0.051	17007.5	17181.63
Partial second-order	545.9	185	2.95	0.901	0.888	0.084	[0.076, 0.092]	0.052	17013.14	17180.01
Two-factor	746	188	3.97	0.847	0.829	0.103	[0.096, 0.111]	0.062	17207.24	17363.22

The results of the model comparisons are presented in Table 4. The correlated four-factor model showed an acceptable, although not uniformly strong, fit to the data, χ^2^(183) = 536.27, χ^2^/df = 2.93, CFI = 0.903, TLI = 0.889, RMSEA = 0.083, 90% CI [0.075, 0.092], and SRMR = 0.051. The partial second-order model, in which AE, OL, and PI loaded onto a common higher-order factor while TR remained a separate first-order factor, showed broadly comparable fit, χ^2^(185) = 545.90, χ^2^/df = 2.95, CFI = 0.901, TLI = 0.888, RMSEA = 0.084, 90% CI [0.076, 0.092], and SRMR = 0.052. Relative to the four-factor model, the changes in CFI (ΔCFI = −0.002) and RMSEA (ΔRMSEA < 0.001) were small. Although the partial second-order model did not improve overall model fit, its standardized higher-order loadings were substantial for AE (β = 0.954), OL (β = 0.872), and PI (β = 0.940), indicating considerable shared variance among these three dimensions. The higher-order factor was also positively associated with TR (r = 0.717).

In contrast, the two-factor model, in which all AE, OL, and PI items were specified to load directly onto a single first-order factor while TR was retained as a separate factor, showed substantially poorer fit, χ^2^(188) = 746.00, χ^2^/df = 3.97, CFI = 0.847, TLI = 0.829, RMSEA = 0.103, 90% CI [0.096, 0.111], and SRMR = 0.062. Its AIC and BIC were also markedly higher than those of the other two models. These results indicate that the strong associations among AE, OL, and PI do not justify collapsing them into a single first-order dimension.

Taken together, the findings support retaining the correlated four-factor structure as the primary measurement representation. At the same time, the partial second-order model provides a plausible supplementary representation of the substantial covariance shared by AE, OL, and PI. This higher-order representation should therefore be interpreted as evidence of commonality among these three dimensions rather than as evidence that all four dimensions form a single general higher-order Digital Anxiety factor.

## Discussion

The present study contributes to the literature by demonstrating that digital anxiety in later life is not adequately captured by existing unidimensional or loosely defined constructs. Existing research indicates that older people’ technology anxiety has remained terminologically fragmented and insufficiently connected to digital inclusion (Kim et al., 2023). Rather than treating digital anxiety as an undifferentiated psychological construct, the findings support a structured, multidimensional configuration that reflects how older people differentially experience digital environments. In particular, the results suggest that digital anxiety is not solely rooted in operational difficulty, as emphasized in early computer anxiety research, nor can it be fully explained by generalized concerns about technological change. Instead, the multidimensional pattern observed in the present study is consistent with the view that digital anxiety is associated with both perceived characteristics of digital environments (e.g., complexity, uncertainty, and opacity) and individuals’ subjective evaluations of their capability within these environments.

This perspective extends technostress theory (Ayyagari et al., 2011) in a non-trivial way. While prior research conceptualizes techno-stressors as properties of digital environments (e.g., complexity, insecurity, uncertainty), the present findings are consistent with the possibility that technology-related demands may be associated with qualitatively different forms of anxiety in later life. Whereas recent conceptual work on “digital phobia” has stressed the diffuse and expanding psychological burden of digital environments, the present study contributes a more delimited and psychometrically validated structure for empirical research. The relatively strong association between operational learning anxiety and ability-based exclusion anxiety indicates that task-related anxiety and competence-related self-doubt are closely connected. This association is consistent with the possibility that difficulties in digital interaction and negative self-evaluations may be related to one another, but the present cross-sectional data cannot establish their temporal ordering or a developmental progression from one form of anxiety to another. The findings therefore support conceptual relatedness between these dimensions rather than a process model of how one develops into the other. Furthermore, the distinction between technological risk anxiety and privacy and institutional anxiety further illustrates the importance of separating outcome-oriented concerns from system-level perceptions. Although the two dimensions are conceptually adjacent and some digital situations may evoke both forms of anxiety, their primary focus differs. Technological risk anxiety concerns anticipated adverse consequences of digital technology use, whereas privacy and institutional anxiety concerns the transparency, trustworthiness, and controllability of the systems and organizations through which digital activities are conducted. The distinction should therefore be understood as relative rather than absolute, reflecting different focal sources of anxiety within interconnected digital experiences.

Taken together, the findings support a multidimensional representation of digital anxiety in later life, with four related but distinguishable domains. The observed correlations among the dimensions, together with the supplementary partial second-order model, suggest that some dimensions may share broader common variance. However, the present cross-sectional data do not establish a developmental sequence, causal mechanism, or underlying psychological hierarchy among these dimensions. The higher-order representation should therefore be understood as a statistical and theoretical account of covariance among selected dimensions rather than as evidence of a demonstrated hierarchical psychological process.

### Theoretical contributions

This study contributes to the literature on digital anxiety and ageing by providing evidence for a multidimensional rather than unidimensional representation of digital anxiety. The correlated four-factor model was retained as the primary measurement representation because it preserved the distinctions among operational learning anxiety, technological risk anxiety, ability-based exclusion anxiety, and privacy and institutional anxiety. At the same time, the relatively strong associations among AE, OL, and PI motivated examination of a partial second-order model. This alternative model showed broadly comparable, but not superior, fit to the correlated four-factor model, and the three dimensions exhibited substantial standardized loadings on the higher-order factor. The result therefore suggests that the covariance among AE, OL, and PI can be represented by a broader common component while preserving their domain-specific structure.

This higher-order representation should be interpreted cautiously. It does not demonstrate that all four dimensions constitute a single hierarchical psychological construct, because TR was not specified as an indicator of the higher-order factor. Nor does the present cross-sectional design establish that the higher-order component reflects a particular causal or developmental mechanism. In particular, perceived control was not measured as an independent construct and therefore cannot be identified as the psychological mechanism underlying the shared variance. The partial second-order model is better understood as a theoretically plausible representation of covariance among AE, OL, and PI that warrants further examination in longitudinal and independent samples.

### Practical implications

The findings suggest that digital anxiety requires interventions beyond skill training alone. Digital literacy may represent a relevant intervention target for operational learning anxiety, although its effectiveness in reducing anxiety requires direct evaluation in intervention studies. Therefore, digital education programs for older people should incorporate components that enhance confidence, clarify system rules, and reduce perceived risks. Second, the results highlight the importance of user-centered design in digital systems. Simplifying interfaces, stabilizing update processes, and reducing unnecessary complexity may represent potentially relevant strategies for addressing operational anxiety, but their effects should be tested directly. At the same time, increasing transparency in data use, improving user control over system operations, and clarifying platform governance may represent relevant targets for addressing privacy and institutional anxiety. Finally, the identification of ability-based exclusion anxiety suggests that perceived capability and confidence should be addressed alongside skill training. Interventions that promote supportive learning contexts, reduce stigma associated with digital incompetence, and encourage intergenerational assistance warrant examination as possible approaches to addressing the social consequences associated with digital anxiety. The structural barriers may be addressed through changes in system design, accessibility, and institutional practices, whereas person-level psychological constraints may require interventions aimed at strengthening confidence, reducing anxiety, and enhancing users’ perceived control over digital interactions.

The findings also speak to frailty prevention by drawing attention to digital anxiety as a potentially consequential feature of older people’s environments. When essential services and opportunities for participation are mediated by digital systems, anxiety about using those systems may restrict access to resources that support everyday functioning and social connection. The four dimensions identified here point to distinct intervention targets: accessible instruction and system design may address operational learning anxiety; transparent safeguards may reduce technological-risk concerns; supportive learning contexts may counter ability-based exclusion; and clearer data practices may strengthen trust in digital institutions. Integrating these targets into digital inclusion initiatives may help create conditions that support older people’s continued access to services and participation—concerns central to a broader approach to frailty prevention.

### Limitations and future research

Several limitations should be acknowledged. First, although the scale demonstrated acceptable psychometric properties, a small number of items showed relatively modest factor loadings and the correlations among some dimensions were comparatively high. These findings suggest that the item structure may still benefit from further refinement as the scale is applied in additional samples. Rather than viewing the present version as a final and fixed instrument, we regard it as a basis for continued psychometric development. Second, the study was largely cross-sectional. The observed associations among operational learning anxiety, ability-based exclusion anxiety, and the other dimensions therefore should not be interpreted as evidence of a temporal or developmental sequence. Similarly, although perceived control may provide a theoretically plausible explanation for some of the common variance among the dimensions, it was not directly measured as an independent construct in the present study. Longitudinal research is needed to determine how these dimensions relate to one another over time and whether perceived control or other psychological processes help account for their shared variance. Finally, the present samples were characterized primarily by age and sex, and several potentially important gerontological characteristics were not systematically assessed, including educational attainment, digital literacy, prior experience with digital technologies, and frequency of technology use. These factors may influence digital anxiety independently of chronological age and may also contribute to heterogeneity among older people. Their absence limits the extent to which the present findings can be generalized across older populations with different educational and technological backgrounds. Future validation studies should therefore collect a broader set of demographic and digital-use characteristics and examine whether the scale functions similarly across these subgroups. Future studies should therefore move beyond cross-sectional validation by using prospective designs to examine whether digital anxiety predicts theoretically relevant behavioral and psychosocial outcomes. Such work would provide a stronger test of the scale’s predictive validity and help clarify how digital anxiety matters for older people’ everyday engagement with digital environments.

## Data Availability

The datasets presented in this study can be found in online repositories. The names of the repository/repositories and accession number(s) can be found at: the derived data supporting the findings of this study are available in the DCD repository (DOI: 10.17632/pcf4rgmdk2.1).
